# Biomonitoring the skeletal muscle metabolic dysfunction in knee osteoarthritis in older adults: Is Jumpstart Nutrition® Supplementation effective?

**DOI:** 10.22088/cjim.14.43.590

**Published:** 2023

**Authors:** Ganguly Apurba, Banerjee Sudip

**Affiliations:** 1 Department of Biochemistry, Techno India University, Salt Lake, Kolkata, India

**Keywords:** Knee osteoarthritis, Jumpstart Nutrition®, Dietary supplement, Biomarkers, Symptomatic slow-acting drugs, Skeletal muscle metabolism.

## Abstract

**Background:**

This study aimed to investigate the efficacy of Jumpstart Nutrition® dietary supplement (JNDS) for enhancing the skeletal muscle metabolism and function of older adults with knee osteoarthritis (KOA) by evaluating the biomarkers of aberrant levels of serum tumor necrosis factor-alpha (TNF-α), interleukin-10 (IL-10), C-reactive protein (CRP), creatine kinase-muscle (CK-MM), and aldolase-A (Aldo-A).

**Methods:**

This twelve-week registry included 54 patients treated with JNDS mainly comprised of calcium, phosphorus, vitamin-K_2_, coenzyme-Q_10_, boswellic acid, and curcumin mixed with soy and whey protein (experimental group) and 51 patients treated with symptomatic slow-acting drugs for osteoarthritis (SYSADOA) (control group) for KOA confirmed with radiological images. At week 0 and week 12 for both the groups evaluated, the non-fasting serum levels of TNF-α, IL-10, CRP, CK-MM, and Aldo-A by using appropriate kits.

**Results:**

At week-twelve, the respective values of area under the ROC curves of the studied biomarkers for pooled experimental cohorts were 0.928, 0.907, 0.908, 0.927, and 0.988 having the significance of accuracy (R-square):66.28%, 47.25%, 70.39%, 65.13%, and 68.00%, indicating a satisfactory treatment policy, their mean± SD, and risk ratio, all exhibited highly significant differences (p<0.0001) and KOA-gradation was upgraded between≥2 and ≥3 from≥4 as per the Kellgren-Lawrence scale compared to the control. Fewer patients had to use emergency medications (p<0.05).

**Conclusions:**

Results suggest that JNDS may be effectively used to strengthen the skeletal muscle metabolism and function of elderly patients with KOA confirmed with the stabilization of studied biomarkers as an alternative to the treatment of SYSAD correlated with ROC curves and the Kellgren-Lawrence scale.

Skeletal muscle metabolic dysfunction is a common feature of sarcopenia of aging and chronic diseases such as knee osteoarthritis (KOA) (1). The signaling pathways of nutritional, hormonal, inflammatory, and nervous stimuli control skeletal muscle metabolism and function (2). KOA is a degenerative disease of the articular cartilage matrix, bone, synovium, and periarticular tissue inflammation (3-6). This degenerative disease is characterized by biochemical composition and structure changes, which alter the tissue’s biological and biomechanical functions. The biological changes include loss of matrix protein such as proteoglycan and collagen, an increase in macroscopic degenerative fibrillation, and altered water content (7-9).Therefore, it is the clinical and pathological outcome of a range of disorders that results in structural and functional failure of muscle such as skeletalmuscle atrophy,neuromuscular junctiondegeneration,hormone imbalance, cytokine imbalance,protein synthesis, andproteolysis, especially over the age of 60 (10-12).

The percentage of the elderly population over the age of 60 years in India is projected to reach 19 percent by 2050 from 8 percent in 2015. The elderly will constitute nearly 34 percent of the total population in the country by the end of the century (13). At the same time, the population aged 65 years and above is expected to have reached 89 million by 2050 from 40.5 million in 2010 in the United States. This increasing phenomenon will drastically affect healthcare systems for decades due to the inevitable growing proliferation of chronic age-related diseases such as KOA (14).

Often, a combination of non-pharmacological and pharmacological treatment approaches can manage KOA on an individual basis. The toxicity and adverse event profile of the most commonly used existing treatments (such as non-steroidal anti-inflammatory drugs (NSAIDs), corticosteroids for relieving pain, inflammation, stiffness, improvement of quality of life temporarily, cyclo-oxygenase-2 (COX 2) inhibitors, and total joint replacement) are uncomplimentary (12-13). The emerging evidence has challenged this recommendation and revealed the potential for adverse events (14-15).

There is no cure for KOA in conventional treatment. But primarily reducing pain and improving joint function aimed at the current therapeutic strategies. This new suggestion could hypothetically increase public interest in the welfares of alternative treatments. Currently, 69% of patients with KOA take some form of supplements for their condition as an alternative therapy (16). It has driven expenditures for alternative treatments to nearly equal those spent on traditional pharmacological therapy (17). 

The compounds of glucosamine and chondroitin, in particular, have attracted a great deal of attention due to massive publicity in the press. Because of this publicity, KOA is the leading medical condition for which people use alternative therapies. The first choice of alternative treatment of KOA with symptomatic slow-acting drugs for osteoarthritis (SYSADOA), which include glucosamine sulfate, glucosamine hydrochloride, chondroitin sulfate, hyaluronic acid, avocado soybean unsaponifiables (ASU) (1:2 Avocado and soybean oils), and diacerein are more commonly recommended (18-20). 

Glucosamine and chondroitin seem to have the same benefit as placebo (21), and there is controversy over whether they also have structure-modifying benefits. These drugs may improve patient pain symptoms as well as reduce cartilage degradation, also having decreased occurrence of gastrointestinal (GI) adverse events when compared to NSAIDs (22-23). Nevertheless, patients with KOA use these widely. However, these supplements’ clinical guidelines, safety, and efficacy are still controversial, and their clinical benefits or herms have not been established (24-25).On the other hand, Jumpstart Nutrition® dietary supplement (JNDS) is recommended as the most effective with low-cost alternatives in managing KOA. The JNDS contains the vital minerals (calcium, phosphorus, magnesium, and iron) required for improving bone and muscle health, essential vitamins (coenzyme Q10, vitamin-K2,vitamin-C, folic acid, and vitamin-D2) needed for muscles and bones, antioxidants (boswellic acids and curcumin) for relieving pain, inflammation, and stiffness of muscles and natural protein powers of soy and whey for promoting mainly bone growth and muscle strength, including sarcopenia due to old age for the treatment of KOA. The ingredients used in JNDS were according to their dietary reference intakes approved by the Food Safety and Standards Authority of India (FSSAI) to treat KOA patients (26-27) based on the recommendation of the Food and Nutrition Board of the Institute of Medicine, National Academy of Sciences (Washington, DC, USA).

However, human biomonitoring is the foundation of community public health evaluation, preclinical health effect assessments, pharmacological drug development and testing, and medical diagnostics (28). During KOA, inflammatory stimuli can alter cytoskeletal components of cells in musculoskeletal tissues. The key roles in maintaining cell biological as well as mechanical functions depend on the cytoskeleton of a cell consisting of filamentous actin, intermediate filaments, and tubulin microtubules. Therefore, it is essential to evaluate the effects of inflammation on cellular biology, cellular mechanical properties, and cellular mechanotransduction during KOA. It includes the change in cell mechanobiology and mechanosensitivity under pro-inflammatory conditions that could be the result of altered mechanotransduction pathways and cytoskeletal changes induced by pro-inflammatory cytokines leading to significant changes in cellular biophysical properties (29).

Therefore, to identify the inflammatory status during KOA, the biomarkers such as serum tumor necrosis factor-alpha (TNF-α), interleukin-10 (IL-10), and C-reactive protein (CRP) (30-35) are assessed, as KOA is an inflammatory disease (36). Besides these, to assess muscle degeneration, including muscular dystrophy and skeletal muscle damage during KOA, are recommended to test the other biomarkers such as creatine kinase-muscle (CK-MM), and aldolase-A (Aldo-A) (34, 37-42). In muscle, creatine is phosphorylated (adenosine triphosphate (ATP) consumption, catalyzed by creatine kinase) to phosphocreatine.

This present supplement registry study (JNDS) aimed to conduct a pilot assessment for the treatment of skeletal muscle metabolic dysfunction for elderly patients including inflammation, muscle degeneration, and bone erosion by evaluating the aberrant levels of biomarkers such as TNF-α, IL-10, CRP, CK-MM and Aldo A in the management of KOA compared to the modern alternative treatment with SYSADOA.

## Methods

**Recruitment of Patients: **Out of 342 approached patients, a total of 262 Indian patients of different ethnic groups, aged 45 to 75 years old, suffering for more than six years with KOA were treated at OPTM Health Care (P) Ltd from 1^st^ September 2018 to 30^th^ June 2019 were included in this twelve-week registry. All patients signed an Institutional Review Board-approved consent form for the physical examination, blood sample collection methods, and radiological images required for the study in the first phase of the screening procedure.

**Figure 1:**
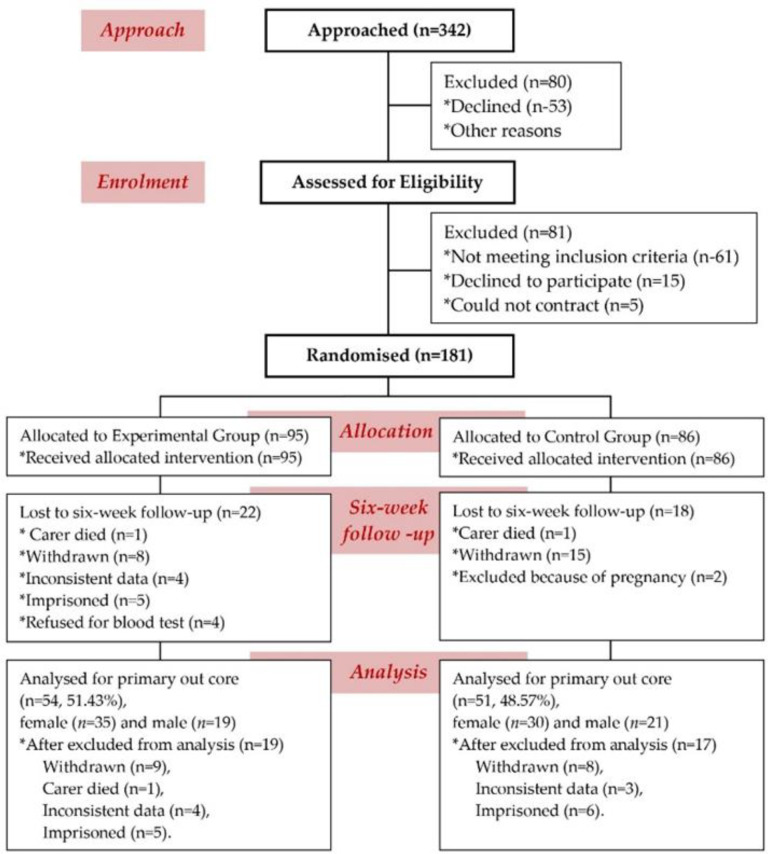
Study flow chart according, to the Consolidated Standards of Reporting Trials (CONSORT), (n = number of subjects)

**Exclusion Criteria: **One hundred fifty-seven of 262 patients were excluded as per protocol adopted in the earlier study (26) and shown in figure 1.

**Study Design**: After evaluating the exclusion criteria, 54 of the remaining 105 patients suffering from acute KOA confirmed with radiological images having severe pain with disabling lifestyle were randomly assigned to the experimental group and treated with JNDS. The remaining 51 subjects were assigned to the control group and treated with symptomatic slow-acting drugs for osteoarthritis (SYSADOA).

 The study flow chart, according to consolidated standards of reporting trials, is shown in figure 1. The demographic data and baseline characteristics of all cohorts are presented in table 1. 

In this study, the drugs belonging to the SYSADOA group to manage uncontrolled index-knee pain are believed to be alleviative medicines. Among them, glucosamine sulfate, chondroitin, and diacerein are regarded as the most potent joint pain reliever and delay joint destruction and cartilage loss, and anti-inflammatory drugs.

The analyses of pre-and post-treatment consumptions of SYSADOA and JNDS made as under: out of 54 patients in the experimental group (JNDS group), 32 patients used only JNDS, and the balanced 22 patients used alleviative medicines mostly glucosamine, chondroitin, diacerein, collagen hydrolysate together with JNDS at the beginning of the treatment. At the end of twelve weeks, only five patients were taking the alleviative medicines plus JNDS in the experimental group. Whereas, in the control group, 30/51patients gulped only glucosamine, chondroitin, diacerein, and collagen hydrolysate and the rest of 21 patients have taken other alleviative medicines such as vitamin D, pycnogenol, methylsulfonylmethane (MSM), and avocado soybean unsaponifiables (1:2 avocado oil and soybean oil) at the beginning of the treatment and more number of patients had shifted to SYSADOA categories because of the inept performance of other supplements at the end of twelve weeks.

**Table 1:** Demographic data and baseline characteristics of the study subjects

**Characteristic**	**Experimental group**	**Control group**
No of subjects (%)	54 (51.43)	51 (48.57)
Female (%)	35 (64.81)	30 (58.82)
Mean age (SD) in years	61.82 (11.26)	60.89 (11.18)
Mean weight (SD) in kg	71.68 (5.02)	71.78 (6.18)
Mean height (SD) in meter	1.54 (0.94)	1.53 (0.89)
Mean BMI (SD) in kg/m²	30.22 (7.34)	30.66 (7.21)
Mean symptom duration in years (SD)	6.29 (1.92)	6.18 (2.09)
**Indian ethnic group (%)**		
Bengali	19 (35.19)	21 (41.18)
Gujrati	5 (9.26)	4 (7.84)
Marwaree	6 (11.11)	7 (13.73)
Marathi	5 (9.26)	4 (7.84)
Tamil	4 (7.41)	5 (9.80)
Punjabi	8 (14.81)	7 (13.73)
Shindhi	3 (5.56)	3 (5.88)
North East India	4 (7.41)	3 (5.88)
**Dietary habits (%)**		
Vegetarian	25 (46.30)	21 (41.18)
Non- vegetarian	29 (53.70)	30 (58.82)
**Other habits (%)**		
Smoking	13 (24.07)	7(13.73)
Drinking alcohol	11 (20.37)	9 (17.65)
Drinking tea and coffee	15 (27.78)	16 (31.37)
Chewing tobacco	6 (11.11)	6 (11.76)
No other habits	9 (16.67)	13 (25.49)
**Analysis of radiological reports (%)**		
KOA in right knee with osteophytes	12 (22.22)	11 (21.57)
KOA in left knee with osteophytes	15 (27.78)	14 (27.45)
KOA in bilateral knees with osteophytes	27 (50.00)	26 (50.98)
**Work status (%)**		
Employed fulltime	5 (9.26)	6 (11.76)
Employed part time	4 (7.41)	3 (5.88)
Housewife / Home- maker	24 (44.44)	23 (45.10)
Retired	17 (31.48)	16 (31.37)
Self employed	4 (7.41)	3 (5.88)
**Marital status (%)**		
Single	4 (7.41)	2 (3.92)
Married	32 (59.26)	34 (66.67)
Separated	3 (5.56)	2 (3.92)
Divorced	4 (7.41)	1 (1.96)
Widowed	11 (20.37)	12 (23.53)
**Multiple complaints or comorbiditie**s** (%)**		
Constipation	34 (62.96)	35 (68.63)
Acidity and reflux	29 (53.70)	31 (60.78)
Insomnia	19 (35.19)	22 (43.14)
Varicose veins	18 (33.33)	17 (33.33)
Urinary incontinence	21 (38.89)	23 (45.10)
Crepitus during knee flexion	41 (75.93)	43 (84.31)
Morning stiffness (<30 min.)	33 (61.11)	31 (60.78)
**Measures taken to diminish pain and inflammation (%)**		
Knee-cap uses	28 (51.85)	25 (49.02)
Lumbar belt uses	23 (42.59)	19 (37.25)
Paracetamol and NSAID use	41 (75.93)	46 (90.20)
Arthrocentesis (four months ago)	16 (29.63)	18 (35.29)
Use of hyaluronic acid injection	19 (35.19)	21 (41.18)
Use of corticosteroid injection	25 (46.30)	31 (60.78)
Massage with herbal or other gels	35 (64.81)	46 (90.20)
Homeopathic treatment	51 (94.44)	48 (94.12)
Ayurvedic treatment	52 (96.30)	49 (96.08)
Stick/walker use	23 (42.59)	21 (41.18)

KOA: knee osteoarthritis

NSAID: Nonsteroidal Anti-Inflammatory Drug

**Evaluation of biochemical parameters:** The collected non-fasting blood samples were centrifuged at 1000×g for 10 min at 4 C to obtain the serum. The biochemical parameters of TNF-α and IL-10 in the blood of patients were measured by ELISA (enzyme-linked immune sorbent assay) method using the kits from R&D System, Germany (Cat. # DY210 and DY217B) (30). The rest of the chemicals was used in laboratory grade supplied by Sigma (St. Louis, MO, USA). 

The methods and protocols were elaborated in detail in the previous studies (34) for analyzing the biomarkers of CRP, CK-MM, and Aldo-A levels for each subject of both groups. The subjects suffering from KOA with inflammation, muscle weakness, and disc degeneration were studied to identify specific biochemical parameters, such as IL-10, TNF-α, CRP, CK-MM, and Aldo-A levels in the affected population.

The mean, standard deviation (SD) and their *p*-values and the values of maximum, minimum, and quartiles of the biomarkers such as IL-10, TNF-α, CRP, CK-MM, and Aldo-A respectively were shown in box and whisker plot for each cohort of both groups. The percentages of improved/retrograded levels of experimental subjects compared to the control subjects were graphically assessed. While analyzing the ROC curves the sensitivity, specificity, significance of accuracy (R-square), cut-off values, and areas under the ROC curves for each biochemical parameter were also evaluated and thereafter, positive predictive value (PPV), negative predictive value, and accuracy were calculated. The risk ratios of the inflammatory biomarkers (CRP, IL-10, and TNF-alpha), muscle degenerative biomarker CK-MM), and bone erosion biomarker (Aldolase-A) for female-only, man-only, and pooled groups were evaluated between both the groups at week 12. The BS-240 Mindray fully automated biochemistry analyzer was used to recheck the final reports of all the biochemical parameters for each cohort of the experimental and the control groups

**Evaluation of radiological images with the Kellgren-Lawrence Grading Scale:** The radiological images for both knee joints of 105 combined-sex patients were collected, both anterior-posterior (AP) and lateral views, at pre- and post-treatment evaluations. The AP views of the knee joints of 105 patients were assessed by the Kellgren-Lawrence (K-L) grading scale (43). The AP view of knee joint x-ray images of two such patients (before and after the treatment) are separately evaluated for each group and depicted in figure 6. 

## Results

**Enrolment and baseline characteristics of patients: **Recruitment started on the 1st of September, 2018. The last follow-up data were received on the 30th of June, 2019. Two-hundred-sixty-two patients were screened out of 342 approaches. A total of 105 patients met the inclusion criteria and were enrolled. Finally, 54 patients were selected for the experimental group, and 51 were in the control group (figure 1).

Different types of food have serious evolvement for eroding our joints and cogging our arteries. In India, people with different ethnic groups have different types of food habits and take different kinds of food at an old age. They are also suffering from other disorders and seek the help of different types of orthotics in KOA. Moreover, according to the research of the Institute of Immunology and Rheumatology, a vegetarian diet resulted in better grip strength and much less pain, joint swelling, tenderness, and morning stiffness. Therefore, in the baseline data analyses, Indian ethnic groups, food habits, other habits such as smoking, and multiple complaints are taken into consideration and shown in table 1.

**Analyses of **alleviative medicines: Analyses of alleviative medicines (SYSADOA), as described above, are taken into consideration at the beginning of the treatments. The decreasing and increasing features of the same for both the groups at the end of twelve-week are shown in table 2. The alleviative medicines especially glucosamine and chondroitin have been diminished by 77.27 % (*p*<0.05) at the end of twelve weeks in the experimental group compared to the increasing number in the control group by 115.19 % (table 2).

**Table 2:** Patients using alleviative medicines (symptomatic slow-acting drugs for osteoarthritis) and Jumpstart® nutrition dietary supplement (JNDS) in the experimental group (*n* = 54) and control group (*n* = 51) at the baseline and twelve weeks

**A) Number of patients used alleviative medicines (SYSADOA) and Jumpstart® nutrition dietary supplement in the experimental group of 54 patients**				
**SUPPLEMENT**	**Baseline (week-0)**		**At week-twelve**	
	**Number**	**%**	**Number**	**%**
JNDS	32	59.26	49	90.74
JNDS + Glucosamine	6	11.11	2	3.70
JNDS + Chondroitin	5	9.26	2	3.70
JNDS + Diacerein	4	7.41	1	1.85
JNDS + Collagen hydrolysate	3	5.56	0	0.00
JNDS + Glucosamine+ Chondroitin	4	7.41	0	0.00
**B) Number of Patients used alleviative medicines (SYSADOA) in the control group of 51 patients**				
**SUPPLEMENT**	**Baseline (Week-0)**		**At week-twelve**	
	**Number**	**%**	**Number**	**%**
Glucosamine	8	15.69	15	29.41
Chondroitin	6	11.76	13	25.49
Glucosamine+ Chondroitin	5	9.80	13	25.49
Diacerein	5	9.80	6	11.76
Collagen hydrolysate	6	11.76	2	3.92
Vitamine D	6	11.76	1	1.96
Pine bark extract (Pycnogenol®)	6	11.76	1	1.96
Methylsulfonylmethan	5	9.80	0	0.00

**Inflammation and muscle degeneration and skeletal muscle damage including bone erosion-related biomarkers: **The mean levels of inflammation-related biomarkers (TNF-α, IL-10, CRP) of the patients of the experimental group at week twelve were highly significant (p<0.0001) compared to the control group, while muscle degeneration and skeletal muscle damage-related biomarkers (CK-MM and Aldo-A) of the patients of the control group were not significant compared to the experimental group (table 3).

**Figure 2:**
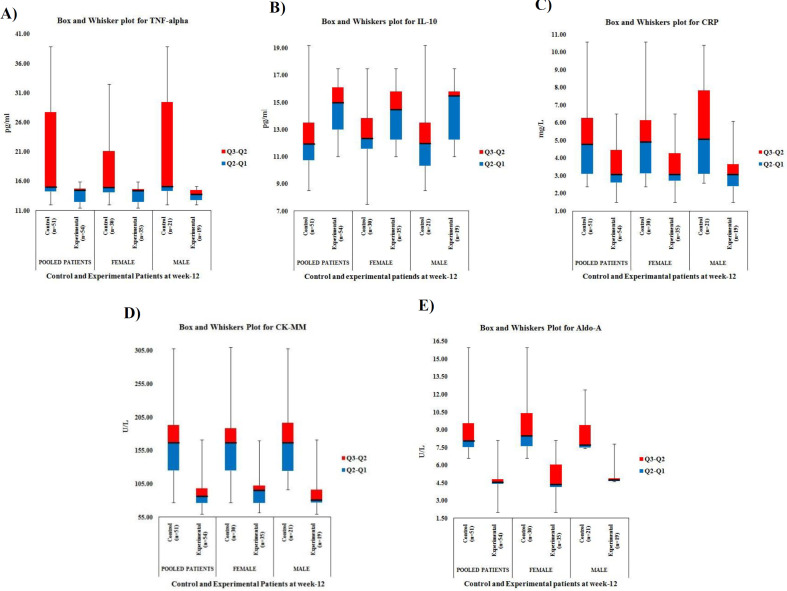
Box and Whisker plots of TNF-α (A), IL-10 (B), CRP (C), CK-MM (D) and Aldo-A (E) of patients for control and experimental groups at week-twelve

Figure 2 shows the Box and Whisker plots for the analyses of control group (n=51) and experimental group (n=54) at week 12 according to gender participation, wherein, it reveals that the values of all biomarkers of the participants of the experimental group were all noted to be highly effective in terms of skewness, interquartile range (IQR) and the respective medians of the patients in the experimental group compared to the control group (table 4).

**Figure 3:**
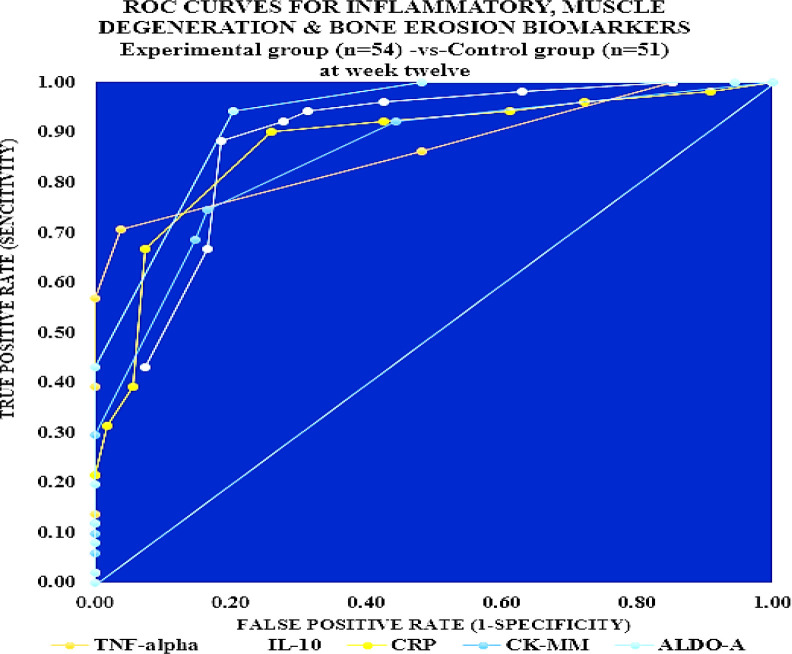
ROC curves for inflammatory, muscle degeneration & bone erosion biomarkers for Experimental group (n=54) -vs-Control group (n=51) at week twelve

The analysis of receiver operating characteristic (ROC) curves in respect of all biomarkers for 51 control subjects and 54 experimental subjects with all significant values of percentage of R-squares, their cut-off points, sensitivity and specificity, areas under the ROC curves (AUC), and the threshold values of all biomarkers are shown in table 5. The ROC curves for the biomarkers for patients of the experimental group versus the control group at week 12 is depicted in figure 3. 

**Figure 4:**
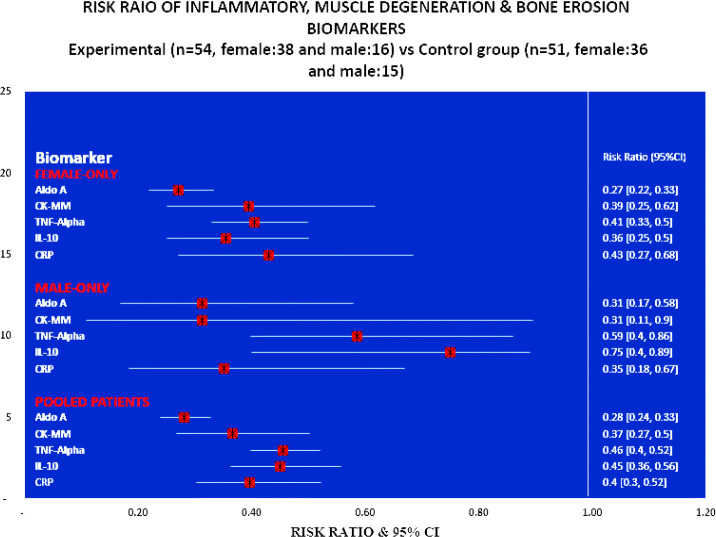
Forest plot showing the Risk ratio of Inflammation, muscle degeneration and bone erosion biomarkers of experimental (n=54, female:38, and male:16 vs Control (n=51, female:36 and male:15) groups at week twelve

**Figure 5:**
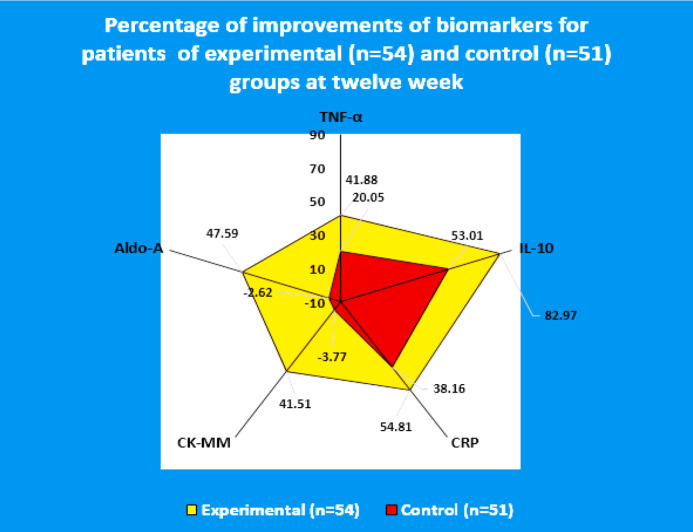
Radar chart showing the percentage of improvements of biomarkers for patients of the Experimental (n=54) and Control (n=51) groups at week-twelve

Figure 4 shows that the risk ratios for all the studied biomarkers for the experimental subjects (female and male) treated with JNS were highly significant (p<0.0001) compared to the control subjects. Radar charts for the percentage of improvements of biomarkers in the control group (n=51, female: 30, male:21) and experimental group (n=54, female:35, male:19) at week-12 over the baseline according to gender participating shows the higher developments, so far as the patients treated with JNDS compared to control group (figure 5). Table 6 shows the strong positive or negative linear relationship between two biomarkers of the experimental and control patients at week twelve.

**Table 3:** Changes in the control group and the experimental group from week-0 to week-12 and changes in control-vs-experimental groups at week-twelve

**Biomarker**	**Gender**	**Changes in control group from week 0-twelve (F**_n_ **: 30, M**_n_ **: 21)**		**Changes in experimental group from week 0-twelve** **(F**_n_ **: 35, M**_n_ **: 19)**		**Changes in control-vs- experimental group at week-twelve**	
		**MD (95% CI)**	**p-value**	**MD (95% CI)**	**p-value**	**MD (95% CI)**	**p-value**
**TNF-α (pg/mL)**	**Female**	-7.06 (-10.25, -3.87)	<0.0001	-12.33 (-14.67, -9.99)	<0.0001	-4.89 (-7.03, -2.75)	<0.0001
	**Male**	-2.79 (-8.29, 2.71)	0.3115	-10.65 (-14.17, -7.13)	<0.0001	-7.73 (-11.89, -3.57)	0.0006
**IL-10 (pg/mL)**	**Female**	5.38 (3.95, 6.81)	<0.0001	7.11 (5.88, 8.34)	<0.0001	1.89 (0.64, 3.13)	0.0035
	**Male**	4.93 (2.77, 6.89)	<0.0001	7.236 (5.21, 9.25)	<0.0001	2.05 (0.37, 3.73)	0.0183
**CRP (mg/L)**	**Female**	-3.12 (-4.34, -1.90)	<0.0001	-4.75 (-5.95, -3.55)	<0.0001	-1.71 (-2.69, -0.73)	0.0009
	**Male**	-3.44 (-5.05, -1.82)	0.0001	-4.91 (-6.05, -3.77)	<0.0001	-1.69 (-3.08, -0.29)	0.0189
**CK_MM (U/L)**	**Female**	6.80 (-10.51, 24.11)	0.435	-76.52 (-95.77, -57.27)	<0.0001	-84.61 (-102.07, -67.11)	<0.0001
	**Male**	6.00 (-22.54, 34.54)	0.6732	-76.00 (-97.21, -54.79)	<0.0001	-82.92 (-108.31, -57.53)	<0.0001
**Aldo-A (U/L)**	**Female**	0.23 (-1.11, 1.57)	0.7331	-4.99 (-6.46, -3.51)	<0.0001	-5.29 (-6.36, -4.22)	<0.0001
	**Male**	0.26 (-1.06, 1.58)	0.6926	-4.54 (-5.56, -3.62)	<0.0001	-4.45 (-6.54, -3.36)	<0.0001
**IL-10:TNF-α**	**Female**	0.48 (0.36, 0.60)	<0.0001	0.75 (0.66, 0.84)	<0.0001	0.31 (-0.17, 0.43)	<0.0001
	**Male**	0.35 (0.15, 0.55)	0.0008	0.70 (0.55, 0.85)	<0.0001	0.36 (0.16, 0.57)	0.00006

MD: mean difference; CI: confidence interval; THF-α: tumor necrosis factor-alpha;IL-10: interlukin-10; CRP: c-reactive protein; CK_MM: creatine kinase-muscle; Aldo-A: aldolase-A; Fn : number of female patient; Mn: number of male patient

**Table 4:** Box and Whisker plot of patients for Control and Experimental groups at week-12

**Parameter**	**Group**	**TNF-alpha** **(pg/ml)**	**IL-10 (pg/ml)**	**CRP** **(mg/L)**	**CK-MM (U/L)**	**Aldo-A (U/L)**
**Mean (SD)**	Control(n=51)	19.92 (7.51)	12.43 (2.80)	5.29 (2.52)	175.45 (72.11)	9.06 (2.15)
	Experimental (n=54)	13.90 (1.24)	14.44 (2.15)	3.69 (1.41)	97.38 (33.64)	5.05 (1.47)
**Median (Q2)**	Control(n=51)	15.00	11.95	4.80	167.00	8.10
	Experimental (n=54)	14.50	15.00	3.11	87.00	4.60
**Range (Maximum-Minimum)**	Control(n=51)	26.90	10.70	8.23	231.00	9.40
	Experimental (n=54)	4.40	6.50	5.00	111.00	6.10
**Q2-Q1**	Control(n=51)	0.7	1.2	1.7	41.75	0.57
	Experimental (n=54)	2	2	0.51	26	0.2
**Q3-Q2**	Control(n=51)	12.7	1.55	1.48	11	1.45
	Experimental (n=54)	0.25	1.1	1.34	11	0.2
**Inter quartile range (Q3-Q1)**	Control(n=51)	13.40	2.75	3.17	67.75	2.03
	Experimental (n=54)	2.25	3.10	1.85	22.00	0.40
**Value of skewness: (Q3-2Q2+Q1)/(Q3-Q1)**	Control(n=51)	0.90	0.13	-0.07	-0.10	0.43
	Experimental (n=54)	-0.78	-0.29	0.45	-0.23	0.00

**Table 5:** Analysis of receiver operating characteristic (ROC) curves for Inflammatory, Muscles degeneration and Bone health biomarkers of experimental group (n=54) vs Control group (n=51) at week

**Study parameters**	**TNF-α(pg/ml)**	**IL=10 (pg/ml)**	**CRP (mg/L)**	**CK-MM (ULL)**	**Aldo-A (U/L)**
**Logistic line of regration**	Y=0.8099x+0.2982	0.4091x+0.6865	0.6765x+0.4538	0.8985x+0.2926	0.9225x+0.2359
**R-square**	0.6628	0.4725	0.7039	0.6513	0.68
**AUC**	0.928	0.907	0.908	0.927	0.988
**Youden's Index (J)**	66.88%	62.64%	64.27%	57.84%	73.75%
**Sensitivity (Sn)**	70.59%	94.12%	90.20%	74.51%	94.12%
**Specificity (Sp)**	96.30%	68.52%	74.07%	83.33%	79.63%
**PPV**	100.00%	21.05%	80.95%	66.67%	100.00%
**NPV**	65.06%	45.35%	59.52%	56.79%	71.05%
**Accuracy**	72.38%	40.95%	63.71%	59.05%	79.05%
**Cut off value**	<18.5pg/ml	>11.9pg/ml	<6.0mg/L	168 U/L	<7.6U/L%

TNF-α: Tumor necrosis factor-alpha; IL-10: Interleukin-10; CRP: C-reactive protein; CK-MM: Creatine kinase-muscle; Aldo-A; Aldolase-A; Y: Linear line of regression; AUC: Area under the curve.

**Table 6:** Correlation coefficients between two biomarkers of the experimental and control patients at week-twelve

		**EXPERIMENTAL GROUP**									
		**Aldo-A**		**CK-MM**		**IL-10**		**TNF-α**		**CRP**	
		**r-value**	**p-value**	**r-value**	**p-value**	**r-value**	**p-value**	**r-value**	**p-value**	**r-value**	**p-value**
**Aldo-A**	**Female**			-0.204	0.239	0.072	0.686	-0.081	0.643	-0.171	0.324
	**Male**			0.271	0.261	-0.154	0.529	0.103	0.675	-0.033	0.894
	**Combined**			-0.222	0.107	0.063	0.651	-0.024	0.864	-0.073	0.597
**CK-MM**	**Female**					-0.077	0.658	0.133	0.005	-0.236	0.171
	**Male**					-0.641	0.003	0.326	0.172	-0.048	0.843
	**Combined**					-0.261	0.057	0.181	0.191	-0.167	0.229
**IL-10**	**Female**							-0.134	0.441	0.328	0.054
	**Male**							-0.097	0.693	0.087	0.723
	**Combined**							-0.111	0.422	0.255	0.063
**TNF-α**	**Female**									0.208	0.229
	**Male**									0.255	0.291
	**Combined**									0.236	0.085
		**CONTROL GROUP**									
**Aldo-A**	**Female**			-0.091	0.635	0.042	0.825	0.016	0.931	-0.047	0.805
	**Male**			0.171	0.457	0.269	0.237	0.005	0.983	0.081	0.726
	**Combined**			0.027	0.849	0.085	0.563	-0.013	0.926	0.022	0.879
**CK-MM**	**Female**					-0.053	0.779	0.032	0.866	-0.044	0.816
	**Male**					-0.182	0.431	-0.429	0.052	0.224	0.329
	**Combined**					-0.109	0.447	-0.217	0.126	-0.133	0.351
**IL-10**	**Female**							-0.464	0.009	0.011	0.955
	**Male**							-0.252	0.269	-0.269	0.238
	**Combined**							-0.418	0.002	-0.144	0.311
**TNF-α**	**Female**									-0.033	0.861
	**Male**									0.099	0.669
	**Combined**									0.057	0.689

TNF-α: Tumor necrosis factor-alpha; IL-10: Interleukin-10; CK-MM: Creatine kinase-muscle; Aldo-A; Aldolase-A.

**Table 7:** Kellgren-Lawrence (KL) grading scale for knee-osteoarthritis for the experimental and the control groups

**Knee joints **	**Gradation**	**Control Group (** ***n*** **=51)**				**Experimental Group (** ***n*** **=54)**			
		**Baseline**		**After twelve-week**		**Baseline**		**After twelve-week**	
		**Number**	**%**	**Number**	**%**	**Number**	**%**	**Number**	**%**
**KOA (Rt. knee)**	Grade-0	None	None	None	None	None	None	None	None
	Grade-1	None	None	None	None	None	None	3	5.55
	Grade-2	None	None	None	None	None	None	5	9.26
	Grade-3	24	47.06	19	37.25	26	48.15	31	57.41
	Grade-4	27	52.94	32	62.75	28	51.85	15	27.78
**KOA (Lt. knee)**	Grade-0	None	None	None	None	None	None	None	None
	Grade-1	None	None	None	None	None	None	2	3.70
	Grade-2	None	None	None	None	None	None	6	11.11
	Grade-3	23	45.10	18	35.29	25	46.30	27	50.00
	Grade-4	28	54.90	33	64.71	29	53.70	19	35.19

Grade 0: no radiographic features of OA are present; Grade 1: doubtful joint space narrowing (JSN) and possible osteophytic lipping; Grade 2: definite osteophytes and possible JSN on anteroposterior weight-bearing radiograph; Grade 3: multiple osteophytes, definite JSN, sclerosis, possible bony deformity; Grade 4: large osteophytes, marked JSN, severe sclerosis and definite bony deformity; n = number of subjects, KOA: Knee osteoarthritis*; n*=number of patients.

**Figure 6:**
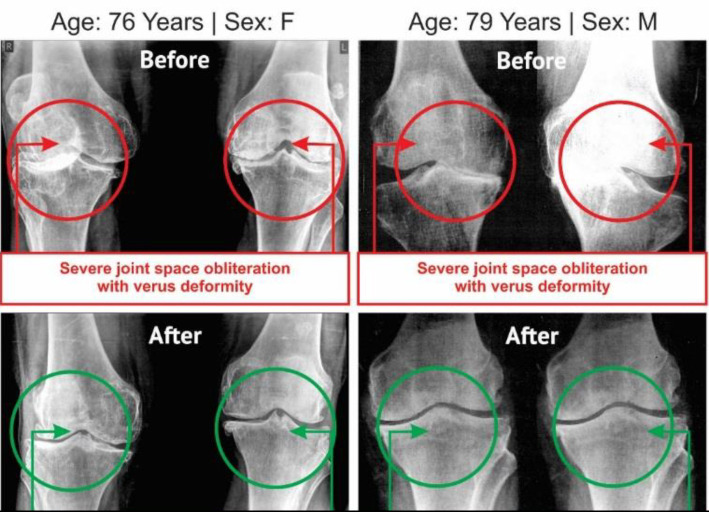
Radiological images of knee joints (one-female and one-male) showing before and after the treatment with JNDS (experimental patients)

**Figure 7:**
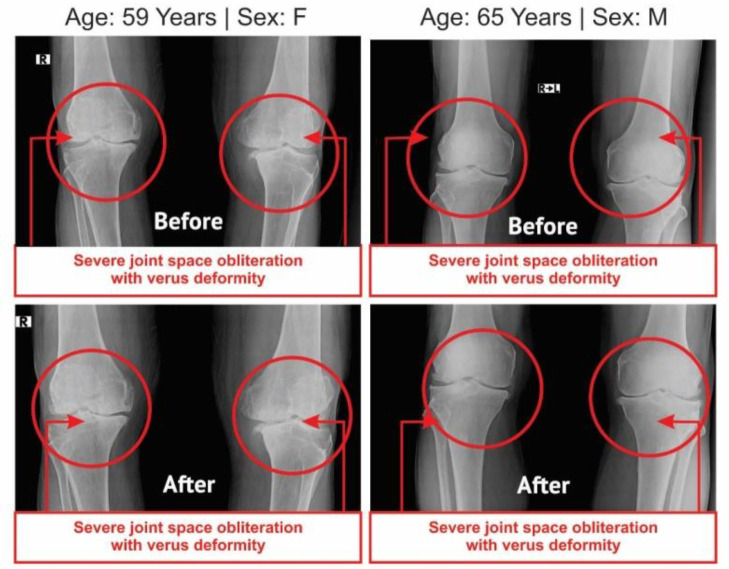
Radiological images of knee joints (one-female and one-male) showing before and after the treatment with SYSADOA (control patients)

**Improvements in bone health as per radiological images as assessed by the Kellgren-Lawrence Scale: **The anterior-posterior (AP) views of the x-ray reports of 105 patients with KOA at the baseline exhibited degenerative changes, particularly in the medial tibiofemoral compartment, with marked joint space narrowing with osteophytes and bilateral varus/valgus deformities. The AP view of x-rays for bilateral knee joints of 54 patients after twelve weeks of treatment with JNDS showed substantial improvements in degenerative changes, as well as bone health, and the balance of 51 patients treated with SYSADOA mainly composed of glucosamine and/or chondroitin, exhibited further deterioration on bone health and assessment under the KL grading scale shown in table 7. The x-ray images of two such patients, female (n = 1) and male (n = 1), before and after the treatment with and without using JNDS, are depicted in figure 6 and figure 7, respectively.

**Safety and cost evaluation: **The safety, tolerability, and comparative cost including treatment, diagnostic, and loss of working days of using JNDS were evaluated as peraprevious study (36).

## Discussion

To the best of our knowledge, this is the first randomized controlled trial to investigate the efficacy of the treatment of skeletal muscle metabolic dysfunction for older adults with KOA, including inflammation, muscle degeneration, and bone erosion using JNDS of the experimental subjects in comparison with the subjects of the control group undergoing SYSADOA confirmed with the biochemical risk factors of aberrant levels of TNF-α, IL-10, CRP, CK-MM and Aldo-A and radiological images.

Several researchers have established that there is a close association between skeletal metabolic dysfunction and the risk factors of inflammation, muscle degeneration, and skeletal muscle damage in KOA. And that the analyses of biomarkers such as TNF-α, IL-10, CRP, CK-MM, and Aldo A can identify the same. (34-47).The reference value for each biomarker in adults has been established as <15 pg/ml TNF-α (30), >12 pg/ml IL-10 (31), <6 mg/L CRP (32), <168 U/L CK-MM (41, 46), and <7.6 U/L Aldo-A (37). 

In this present study, the results show that 59.26% of patients of the experimental group used only JNDS, and the balanced 40.74% used JNDS plus glucosamine, or chondroitin sulfate or diacerein or in the combination of glucosamine and chondroitin at the beginning of the treatment and the end of twelve weeks, they have reduced (9.26%) the other alleviative medicines especially glucosamine, or chondroitin sulfate or diacerein and increased the consumption of JNDS (90.74%) (*p *<0.05) (Table 2). 

At the same time, it observes 47.06% of patients in the control group used glucosamine, chondroitin sulfate, and diacerein (SYSADOA,) and 52.94% of the patients used other alleviative medicines (table 2) at the beginning of the treatment. After that, at the end of twelve weeks, a large number of patients (92.15%) were recommended to increase the dosages of glucosamine or chondroitin, or diacerein to control their increased pain syndrome and disabilities temporarily during KOA (table 2). These results indicate that the efficacy of the treatment with JNDS for KOA elderly patients may be a better option than the modern supplementary treatment with SYSADOA.

Moreover, it is established that KOA is an inflammatory disease (36). Schaap*et al.* (48) have demonstrated that inflammatory markers are strongly associated with the age-related deterioration of skeletal muscle. The major factors responsible for the development of inflammatory processes involved in KOA are aging, sex differences, obesity, joint overuse, joint trauma, surgical intervention, and other systemic factors. Inflammatory pain may be caused by synovitis, bone erosions, swollen joint capsule, ligament damage, muscle weakness, joint fusion, and centralized pain during KOA. In this present study, inflammatory biomarkers, TNF-α, IL-10, and CRP were assessed for KOA. Therefore, the study of these biomarkers is suitable to detect the rate of inflammation and the progression of skeletal muscle dysfunction, especially in KOA. Because TNF- α, and IL-10 in blood showed antagonistic mechanisms (30, 31, 49, 50), the action of anti-inflammatory cytokine (IL-10) mainly involves inhibiting the synthesis of inflammatory cytokine (TNF- α).

Moreover, there are many inflammatory biomarkers. The serum levels of CRP also found to be elevated during inflammation in tissues from a standard value of < 6 mg/L for KOA (50-52). To measure the status of inflammation during KOA, we evaluate CRP. Despite this fact, Ganguly (34) has already explained the reasons for not evaluating the other inflammatory markers such as erythrocyte sedimentation rate (ESR) (53), anti-cyclic citrullinated peptide (anti-CCP), rheumatoid factors, and antinuclear antibody (ANA), to identify the inflammatory status during KOA (54-57).

Researchers have proven that the oral supplement of the gum resin of *Boswelliaserrata* extract can significantly reduce the potential inflammatory marker, serum levels of high-sensitive C-reactive protein, inhibit lipopolysaccharide-mediated TNF-α induction in monocytes by direct interaction with IκB kinases and the activation of nuclear factor kappa B (NF-kB) (a potent inducer of chronic inflammation), and improve the knee joint gap and reduce the osteophytes (spur), associated with KOA (58-62).

Again, researchers have established that CK-MM levels elevate from the standard value of <165 U/L in response to muscular dystrophy, connective tissue damage, etc. (34, 35, 40, 41). And Aldo-A levels increase from the standard value of <7.6 U/L due to skeletal muscle damage and bone erosion (34, 40, 41), suggesting that these markers may be the risk factors for KOA. However, Ryall*et al.* (63) observed that the cellular and molecular mechanisms are responsible for age-related skeletal muscle wasting and weakness. These studies have demonstrated that CK-MM and Aldo-A levels are closely related to age-associated connective tissue damage, bone erosion, and skeletal muscle damage. Moreover, Konopka*et al.*(64) and Keller *et al*.(65), have suggested that the vitamin K_2_ (coagulation vitamin) and coenzyme Q_10_, contained in JNDS, are required for muscle dysfunction associated with structural and alteration of skeletal muscle mitochondria, which controls the metabolism of reactive oxygen species (ROS), Ca^ 2+ ^homeostasis, and apoptosis during the aging of skeletal muscles. Several studies have further emphasized that the coenzyme-Q_10_ is essential for the health of virtually all human tissues (epithelial, connective, muscular, and nervous) and organs (66-75) and was well explained in the earlier study (36). 

The percentage of improvement of biomarkers after JNDS therapy has been shown with the help of a Radar chart as the researchers have established that the Radar chart is a potentially effective tool that may be used to communicate value in health care by visually representing outcome and cost data (71). According to Avila et al.(72) and Jones et al.(73), the results of the box and whisker plots as well as ROC curve analysis are useful to understand the differential mechanisms of action of each treatment and could help to explain the differences observed in clinical trials, meta-analyses, and observational studies (tables 4-5 and figures 2-5).

The area under the ROC curve (AUC) for Aldo-A (0.988) was higher followed by TNF-α (0.928), indicating the satisfactory treatment policy so far as the muscle degeneration and skeletal muscle damage including bone erosion. On the other hand, the higher AUCs were obtained for TNF-α (0.928), IL-10 (0.907), and CRP (0.785), indicating satisfactory anti-inflammatory effects was achieved with JNDS according to the international threshold limits of inflammatory biomarkers, and the results of IL-10 show the effects of inflammatory cytokine (table 5 and figure 3.)

Therefore, our results indicate that the treatment with JNDS may be a better option for the effective improvements of muscle degeneration and skeletal muscle damage that occurred during KOA than the conventional supplementary treatment, mostly with the combination of glucosamine and chondroitin or diacerein in the category of SYSADOA.

In previous studies, Ganguly has established that JNDS can ameliorate the calcium-to-phosphorus ratio and parathyroid hormone (26). It also helps to make symmetry of aberrant lower extremities and standardize outcome measures and obesity of patients with osteoarthrosis (27). Moreover, definite improvements observe in reducing osteophytes, joint space narrowing, sclerosis, and bony deformities of knee joints of the KOA patients treated with JNDS, when assessing the KL grading scale. More numbers of patients of the experimental group have shifted to higher grades ( 2 and 3) from grade 4 for both rights and left knee joints with JNDS as against further deterioration of OA in the control group (table 7 and figures 6 and 7). Clinical improvements were detected in inflammation status, muscle degeneration, and bone erosion as evidenced by the analyses of related evidence-based approved biomarkers viz IL-10, TNF-α, CRP, CK-MM, and Aldo-A (figures 2-5).

The focus of this pilot registry study and previous evaluations of KOA patients was mainly on the safety profile and the efficacy of JNDS in reducing and controlling inflammation, improving muscle degeneration, in the management of costs, and in the use of other conventional supplementary products. Considering the tremendous opportunity for using safe products of a natural origin in pharmaceutical standards, particularly in subjects in remission or non-acute phases, JNDS could be an important management option. It may also consider the high social cost of managing these patients whose number is growing with increasing age (61). This supplement registry indicates that the use of JNDS in KOA is effective and has limited costs, which may be used even without a prescription for safe self-medication.

Finally, the results indicate that there is moderate/fair agreement between the inter-rater reliabilities and the degree of accuracy of the data collected for the analyses of biochemical parameters of patients treated with JNDS compare to control subjects as evidenced from the Cohen’s kappa (*k*) values: (*k:*0.58, 95% CI: 0.0.425 to 0.736, moderate agreement) for Aldo-A, (*k:*0.38, 95% CI: 0.217 to 0.551, fair agreement) for CK-MM, (*k*:0.27, 95% CI:0.091 to 0.453, fair agreement ) for TNF-α, (*k*: 0.21, 95 % CI: 0.034 to 0.388, fair agreement) for IL-10, and (*k:* 0.23, 95% CI: 0.064 to 0.389, fair agreement) for CRP.

Therefore, JNDS especially combined with vitamin-K_2_, coenzyme Q _10_, boswellic acids, and curcumin mixed with protein powers of soy and whey, seems to be rapidly effective even in comparison with other supplementary products, as mentioned above, on the signs/symptoms of KOA. It acts quickly in a large number of patients (74) including subjects with a main inflammatory component and those with a degenerative component (associated with different types and levels of pain) along with the maintenance of appropriate levels of biomarkers such as TNF-α, IL-10, CRP, CK-MM, and Aldo-A in their serum.

However, this study has several significant limitations. Firstly, the results evaluated based on a small sample size may be biased. The supplement was studied in the short term (≤3 months), but we are unsure whether equivalent efficacy and safety would have been achieved in the long run. Secondly, the assessment of KOA has been determined based on x-ray images. Still, for better sensitive measures of cartilage pathology, the higher resolution requires magnetic resonance imaging. Conflicting evidence exhibits the association between meniscal subluxation and pain as it is a risk factor for cartilage loss and joint space narrowing in people with symptomatic KOA (75, 76).Thirdly, patients are restricted to treatment with the supplement of those who are suffering from the following disorders: adverse pathogenic effects on milk products; concomitant diseases that required parallel multiple drug treatment; a history of cancer including caranomatosis and granulocytic leukemia; a history of chronic liver, heart and kidney diseases; patients refuse to do x-rays, blood tests, and a physical evaluation and /or attend weekly follow-up visits; patients with dementia, morbid obesity, pregnancy, prior knee surgery, and red flag symptoms. 

Our results suggest that the use of JNDS is the better option as an alternative to the conventional supplementary treatment with SYSADOA for the risk factors of skeletal muscle metabolic dysfunction including inflammation, muscle degeneration, and bone erosion for patients with KOA. The study results further confirmed with normalization of aberrant levels biomarkers such as IL-10, TNF-α, CRP, CK-MM, and Aldo-A (tables 3-6 and figures 2-5 and radiological images (figures 6-7) correlated with KL grading scale (table 7). Further research is suggested to be undertaken on: analyses for more biomarkers and /or imaging biomarkers to achieve enough value to predict KOA progression in the clinic and treatment thereon; phytochemicals characterization of JNDS by using mass spectroscopy; receptor and ligand binding activities through molecular docking to know which compounds are responsible in the normalization of KOA and prevention thereon; estimation of reactive oxygen species (ROS), superoxide dismutase (SOD), catalase (CAT), glutathione oxide and peroxide to prove that free radicals are responsible for KOA and their levels become normal at the end of treatment with JNDS; measurements of collagen and 4-hydroxyproline (O-Hyp) to show collagen formation which takes place with the treatment; measurements of hyaluronic acid both in volume and chemical compositions before and after the treatment to show how the phytochemicals of JNDS changing the chemical compositions of hyaluronic acid, and analyses of dystrophin proteins for muscular dystrophy occurred during KOA and their effects on the treatment with JNDS.

## References

[1] Biolo G, Cederholm T, Muscaritoli M (2014). Muscle contractile and metabolic dysfunction is a common feature of sarcopenia of aging and chronic diseases: from sarcopenic obesity to cachexia. Clin Nutr.

[2] (2019). Nutrition and Skeletal Muscle.

[3] Johnson VL, Hunter DJ (2014). The epidemiology of osteoarthritis. Best Pract Res Clin Rheumatol.

[4] Garnero P, Piperno M, Gineyts E (2001). Cross-sectional evaluation of as biochemical markers of bone, cartilage, and synovial tissue metabolism in patients with knee osteoarthritis: relations with disease activity and joint damage. Ann Rheum Dis.

[5] Benito MJ, Veale DJ, FitzGerald O, van den Berg WB, Bresnihan B (2005). Synovial tissue inflammation in early and late osteoarthritis. Ann Rheum Dis.

[6] Goldring MB, Goldring SR (2010). Articular cartilage and subchondral bone in the pathogenesis of osteoarthritis. Ann N Y Acad Sci.

[7] Armstrong CG, Mow VC (1982). Variations in the intrinsic mechanical properties of human articular cartilage with age, degeneration, and water content. J Bone Joint Surg Am.

[8] Meachim G, Denham D, Emery IH, Wilkinson PH (1974). Collagen alignments and artificial splits at the surface of human articular cartilage. J Anat.

[9] Wong BL, Bae WC, Chun J (2008). Biomechanics of cartilage articulation: effects of lubrication and degeneration on shear deformation. Arthritis Rheum.

[10] Neogi T, Zhang Y (2013). Epidemiology of osteoarthritis. Rheum Dis Clin North Am.

[11] Nuki G (1999). Osteoarthritis: a problem of joint failure. Z Rheumatol.

[12] Wang J, Leung KS, Chow SK, Cheung WH (2017). Inflammation and age-associated skeletal muscle deterioration (sarcopaenia). J Orthop Translat.

[13] United Nations Population Fund Caring for Our Elders: Early Responses’- India Ageing Report–2017. UNFPA, New Delhi, India 201.

[14] Dall TM, Gallo PD, Chakrabarti R (2013). An aging population and growing disease burden will require a large and specialized health care workforce by 2025. Health Aff.

[15] Palo N, Chandel SS, Dash SK (2015). Effects of osteoarthritis on quality of life in elderly population of Bhubaneswar, India, a prospective multicenter screening and therapeutic study of 2854 patients. Geriatr Orthop Surg Rehabil.

[16] Jordan KM, Arden NK, Doherty M (2003). EULAR Recommendations 2003: an evidence-based approach to the management of knee osteoarthritis: report of a task force of the standing committee for international clinical studies including therapeutic trials (ESCISIT). Ann Rheum Dis.

[17] Zhang W, Moskowitz RW, Nuki G (2008). OARSI recommendations for the management of hip and knee osteoarthritis, Part II: OARSI evidence-based, expert consensus guidelines. Osteoarthritis Cartilage.

[18] da Costa BR, Reichenbach S, Keller N (2016). Effectiveness of non-steroidal anti-inflammatory drugs for the treatment of pain in knee and hip osteoarthritis: a network meta-analysis. Lance.

[19] Basedow M, Runciman WB, March L, Esterman A (2014). Australians with osteoarthritis: the use of and beliefs about complementary and alternative medicines. Complement Ther Clin Pract.

[20] Ramsey SD, Spencer AC, Topolski TD, Belza B, Patrick DL (2001). Use of alternative therapies by older adults with osteoarthritis. Arthritis Rheum.

[21] Machado GC, Maher CG, Ferreira PH (2015). Efficacy and safety of paracetamol for spinal pain and osteoarthritis: systematic review and meta-analysis of randomised placebo-controlled trials. BMJ.

[22] Towheed TE, Maxwell L, Anastassiades TP (2005). Glucosamine therapy for treating osteoarthritis. Cochrane Database Syst Rev.

[23] Anson P (2016). Supplements help relieve pain of osteoarthritis.

[24] Liu X, Machado GC, Eyles JP, Ravi V, Hunter DJ (2018). Dietary supplements for treating osteoarthritis: a systematic review and meta-analysis. Br J Sports Med.

[25] Kongtharvonskul J, Anothaisintawee T, Mark McEvoy M (2015). Efficacy and safety of glucosamine, diacerein, and NSAIDs in osteoarthritis knee: a systematic review and network meta-analysis. Eur J Med Res.

[26] Ganguly A (2019). Role of Jumpstart Nutrition®, a Dietary Supplement, to Ameliorate Calcium-to-Phosphorus Ratio and Parathyroid Hormone of Patients with Osteoarthritis. Med Sci.

[27] Ganguly A, Banerjee SK (2023). Analysis of Receiver Operating Characteristic Curve for Biomarkers and Lower Extremities As Predictors of Osteoarthritis Risk. Int J Recent Sci Res.

[28] Pleil JD, Wallace MAG, Stiegel MA, Funk WE (2018). Human biomarker interpretation: the importance of intra-class correlation coefficients (ICC) and their calculations based on mixed models, ANOVA, and variance estimates. J Toxicol Environ Health B Crit Rev.

[29] Nguyen QT, Jacobsen TD, Chahine NO (2017). Effects of inflammation on multiscale biomechanical properties of cartilaginous cells and tissues. ACS Biomater Sci Eng.

[30] Vilcek J, Lee TH (1991). Tumor necrosis factor New insights into the molecular mechanisms of its multiple actions. J Biol Chem.

[31] Gesser B, Leffers H, Jinquan T (1997). Identification of functional domains on human interleukin-10. Proc Natl Acad Sci U S A.

[32] Pepys MB, Weatherall DJ, Ledingham JGG, Warrell DA ( 1995). The acute phase response and C-reactive protein. Oxford textbook of medicine.

[33] Pearle AD, Scanzello CR, George S (2007). Elevated high-sensitivity C-reactive protein levels are associated with local inflammatory findings in patients with osteoarthritis. Osteoarthritis Cartilage.

[34] Ganguly A (2019). Levels of C-reactive protein, creatine kinase-muscle, and aldolase A are suitable biomarkers to detect the risk factors for osteoarthritic disorders: A novel diagnostic protocol. Caspian J Intern Med.

[35] Ganguly A (2018). Efficacy of phytotherapeutic protocol for the risk factor of elevated level of serum C-reactive protein in knee-osteoarthritis: Part I A systematic meta-analysis. Int Arch Bio Med Clin Res.

[36] Bar-Or D, Rael LT, Thomas GW, Brody EN (2015). Inflammatory pathways in knee osteoarthritis: potential targets for treatment. Curr Rheumatol Rev.

[37] Feissli S (1966). Quantitative determination of Aldolase. Klin. Wschr.

[38] Long F, Cai X, Luo W, Chen L, Li K (2014). Role of Aldolase in osteosarcoma progression and metastasis: In vitro and in vivo evidence. Oncol Rep.

[39] Parsons S, Alesci S, Feuerstein G, Wang J (2008). Biomarkers in the development of novel disease-modifying therapies for osteoarthritis. Biomarkers Med.

[40] Ganguly A (2018). Role of topical phytotherapy on the risk factor of elevated level of serum aldolase-A in knee-osteoarthritis: Part II A systematic meta-analysis. Int Arch Bio Med Clin Res.

[41] Ganguly A (2018). Impact of topical phytotherapeutic effects on elevated level of serum creatine kinase-muscle as risk factor of muscular degeneration in knee-osteoarthritis: Part II A systematic meta-analysis. Int Arch Bio Med Clin Res.

[42] Ganguly A (2019). Evaluation of A cost-effective novel diagnostic method for lumbar herniated disc with knee-osteoarthritis: a randomized sample study. Med Sci.

[43] Kellgren JH, Lawrence JS (1957). Radiological assessment of osteoarthrosis. Ann Rheum Dis.

[44] Smith JW, Martins TB, Gopez E (2012). Significance of C-reactive protein in osteoarthritis and total knee arthroplasty outcomes. Ther Adv Musculoskelet Dis.

[45] Kim HJ, Lee YH, Kim CK (2007). Biomarkers of muscle and cartilage damage and inflammation during a 200 km run. Eurp J Appl Physio.

[46] Porter RS, Kaplan JL (2011). The Merck manual of diagnosis and therapy.

[47] Bennell KL, Hunter DJ, Hinman RS (2012). Management of osteoarthritis of the knee. BMJ.

[48] Schaap LA, Pluijm SM, Deeg DJ, Visser M (2006). Inflammatory markers and loss of muscle mass (sarcopenia) and strength. Am J Med.

[49] Idriss HT, Naismith JH (2000). TNF alpha and the TNF receptor superfamily: structure-function relationship(s). Microsc Res Tech.

[50] Wojdasiewicz P, Poniatowski ŁA, Szukiewicz D (2014). The role of inflammatory and anti-inflammatory cytokines in the pathogenesis of osteoarthritis. Mediators Inflamm.

[51] Spector TD, Hart DJ, Nandra D (1997). Low-level increases in serum C-reactive protein are present in early osteoarthritis of the knee and predict progressive disease. Arthritis Rheum.

[52] Rifai N, Tracy RP, Ridker PM (1999). Clinical efficacy of an automated high sensitivity C-reactive protein assay. Clin Chem.

[53] Hashemi R, Majidi A, Motamed H (2015). Erythrocyte sedimentation rate measurement using as a rapid alternative to the Westergren method. Emerg (Tehran).

[54] Pagana K, Pagana TJ (2014). Mosby's manual of diagnostic and laboratory tests.

[55] Merlini AB, Schafransk MD, Mansani FP, Doi: 10.13140/2.1.1017.536 (2014). Anti-Cyclic Citrullinated Peptide (ANTI-CCP) antibodies in different autoimmune diseases. Advances in Medicine and Biology.

[56] Kobak S, Ylmaz H, Sever F, Sen N (2014). Anti-cyclic citrullinated peptide antibodies in patients with sarcoidosis. Sarcoidosis, Vasc Diffuse Lung Dis.

[57] Ali H, Alam J, Nadeem A, Naureen S (2019). Diagnostic accuracy of anti-cyclic citrullinated peptide antibodies in rheumatoid arthritis. Pak J Physicl.

[58] Belcaro G, Dugall M, Luzzi R (2018). Phytoproflex®: Supplementary management of osteoarthrosis: A supplement registry. Minerva Med.

[59] Cuaz-Pérolin C, Billiet L, Baugé E (2008). Anti-inflammatory and antiatherogenic effects of the NF-κB inhibitor acetyl-11-keto-β-boswellic acid in LPS-challenged ApoE−/−mice. Arteriosclerosis Thromb Vasc Biol.

[60] Syrovets T, Büchele B, Krauss C, Laumonnier Y, Simmet T (2005). Acetyl-boswellic acids inhibit lipopolysaccharide-mediated TNF-α induction in monocytes by direct interaction with IκB kinases. J Immunol.

[61] Majeed M, Majeed S, Narayanan NK, Nagabhushanam K (2019). A pilot, randomized, double-blind, placebo-controlled trial to assess the safety and efficacy of a novel Boswelliaserrata extract in the management of osteoarthritis of the knee. Phytother Res.

[62] Pearle AD, Scanzello CR, George S (2007). Elevated high-sensitivity C-reactive protein levels are associated with local inflammatory findings in patients with osteoarthritis. Osteoarthr Cartil.

[63] Ryall JG, Schertzer JD, Lynch GS (2008). Cellular and molecular mechanisms underlying age-related skeletal muscle wasting and weakness. Biogerontology.

[64] Konopka AR, Nair KS (2013). Mitochondrial and skeletal muscle health with advancing age. Mol Cell Endocrinol.

[65] Keller K, Engelhardt M ( 2014). Strength and muscle mass loss with aging process. Age and strength loss. Muscles Ligaments Tendons J.

[66] Brotto M, Abreu EL (2012). Sarcopenia: Pharmacology of today and tomorrow. J Pharmacol Exp Ther.

[67] Cruz-Jentoft AJ, Landi F (2014). Sarcopenia. Clin Med.

[68] Scott D, Blizzard L, Fell J, Giles G, Jones G (2010). Associations between dietary nutrient intake and muscle and strength in community-dwelling older adults: The Tasmanian Older Adult Cohort Study. J Am Geriatr Soc.

[69] Sukkar SG, Bounous G (2004). The role of whey protein in antioxidant defense. Riv Ital Nutr Parenter Enter.

[70] Bulut Solak B, Akin N (2012). Functionality of whey protein. Int J Health Nutr.

[71] Thaker NG, Ali TN, Porter ME (2016). Communicating value in health care using radar charts: a case study of prostate cancer. J Oncol Pract.

[72] Avila M, Alonso A, LόpezLasanta M (2015). AB0425 biological therapies in rheumatoid arthritis: graphical analysis of DAS28 and CDAI components evolution over time. Ann Rheum Dis.

[73] Jones LD, Bottomley N, Harris K (2016). The clinical symptom profile of early radiographic knee arthritis: a pain and function comparison with advanced disease. Knee Surg Sports Traumatol Arthrosc.

[74] Cockburn E, Hayes PR, French DN, Stevenson E, St Clair Gibson A (2008). Acute milk-based protein-CHO supplementation attenuates exercise-induced muscle damage. Appl Physiol Nutr Metab.

[75] Evans W J (2010). Skeletal muscle loss: Cachexia, sarcopenia, and inactivity. Am J Clin Nutr.

[76] Rintelen B, Neumann K, Leeb BF (2006). A meta-analysis of controlled clinical studies with diacerein in the treatment of osteoarthritis. Arch Intern Med.

